# Seroprevalence of antibodies against SARS-CoV-2 and risk of COVID-19 in Navarre, Spain, May to July 2022

**DOI:** 10.2807/1560-7917.ES.2022.27.33.2200619

**Published:** 2022-08-18

**Authors:** Jesús Castilla, Óscar Lecea, Carmen Martín Salas, Delia Quílez, Ana Miqueleiz, Camino Trobajo-Sanmartín, Ana Navascués, Iván Martínez-Baz, Itziar Casado, Cristina Burgui, Nerea Egüés, Guillermo Ezpeleta, Carmen Ezpeleta

**Affiliations:** 1 Instituto de Salud Pública de Navarra, Pamplona, Spain; 2 CIBER Epidemiología y Salud Pública (CIBERESP), Madrid, Spain; 3 Navarre Institute for Health Research (IdiSNA), Pamplona, Spain; 4 Gerencia de Atención Primaria, Servicio Navarro de Salud-Osasunbidea, Pamplona, Spain; 5 Clinical Microbiology Department, Hospital Universitario de Navarra, Pamplona, Spain; 6 Hospital Reina Sofía, Tudela, Spain

**Keywords:** SARS-CoV-2, COVID-19 vaccine, natural immunity, sero-epidemiological survey, anti-N antibodies, Omicron variant

## Abstract

In Navarre, Spain, in May 2022, the seroprevalence of anti-nucleocapsid (N) and anti-spike (S) antibodies of SARS-CoV-2 was 58.9% and 92.7%, respectively. The incidence of confirmed COVID-19 thereafter through July was lower in people with anti-N antibodies (adjusted odds ratio (aOR) = 0.08; 95% confidence interval (CI): 0.05–0.13) but not with anti-S antibodies (aOR = 1.06; 95% CI: 0.47–2.38). Hybrid immunity, including anti-N antibodies induced by natural exposure to SARS-CoV-2, seems essential in preventing Omicron COVID-19 cases.

Immune response induced by natural infection and coronavirus disease (COVID-19) vaccines may prevent new severe acute respiratory syndrome coronavirus 2 (SARS-CoV-2) infections [1], but the differences between both effects are not totally understood [2]. After the pandemic waves involving SARS-CoV-2 Omicron subvariants BA.1 and BA.2 [3], a new epidemic wave, characterised by the predominance of the subvariants BA.4 and BA.5 and by special impact on elderly people, affected Europe in the period from May to July 2022 [4-7].

We aimed to estimate the seroprevalence of antibodies against the nucleocapsid (N) and the spike (S) proteins of the SARS-CoV-2 in the population of Navarre in May 2022 and evaluated the effect of these antibodies on the risk of SARS-CoV-2 infection thereafter through July 2022.

## Study design, setting and information sources

This study analysed data from the enhanced epidemiological surveillance of COVID-19 and a sero-epidemiological survey carried out in Navarre (659,231 inhabitants), Spain. By April 2022, more than 30% of the population of Navarre had had a confirmed diagnosis of infection with SARS-CoV-2, 90% of people aged 5 years or older were fully vaccinated for COVID-19, 58% had had an additional dose, and a second booster dose had been offered to immunocompromised patients [4,8].

A sample of primary healthcare facilities (35/57) voluntarily recruited patients aged 5 years and older whose physicians requested a blood test for reasons unrelated to SARS-CoV-2 infection from 26 April to 3 June 2022. Patients with clinical manifestations of COVID-19 or close contact with a COVID-19 case in the previous 10 days, healthcare workers, nursing home residents and people not resident in the region were excluded. The estimated sampling size of 1,600 individuals to ensure a minimum number of 1,200 participants was distributed in quotas by primary healthcare facilities, sex and age groups to maintain a distribution similar to the regional population by sex, age, geographical area and municipality size [9].

A blood sample of each participant was tested for anti-N and anti-S protein antibodies by specific electrochemiluminescence immunoassays (Elecsys anti SARS-CoV-2 assay, Roche Diagnostics, Germany). Anti-S titres higher than 250 U/mL were considered positive. The criteria for interpretation of serological results are shown in Table 1. COVID-19 vaccination status of participants was obtained from the regional vaccination registry. mRNA and viral vector vaccines were used [8]. Each dose was considered 14 days after administration. Sex, age, country of birth (Spain and others) and major chronic conditions (immunocompromised, other conditions and none) were obtained from healthcare records. During the study period, patients consulting for acute respiratory infection were tested with no relevant change in testing policy. Confirmed COVID-19 diagnoses were obtained from the enhanced epidemiological surveillance. Results of variant-specific PCR in Navarre [4] and sequencing in Spain [5] showed an increasing proportion of the SARS-CoV-2 Omicron subvariants BA.4 and BA.5 from May 2022.

**Table 1:** Interpretation of the serological status of the participants in the COVID-19 seroprevalence survey, Navarre, Spain, May 2022

Anti -N antibody result	Anti -S antibody result	Interpretation
Negative	Negative	*More probably:* People not vaccinated for COVID-19 and without past SARS-CoV-2 infection. *Alternatively:* No immune response or loss of antibodies over time.
Negative	Positive	*More probably:* People vaccinated for COVID-19 without past SARS-CoV-2 infection. *Alternatively:* People vaccinated with a past SARS-CoV-2 infection but no immune response or loss of antibodies.
Positive	Negative	*More probably:* People not vaccinated for COVID-19 with past SARS-CoV-2 infection. *Alternatively:* People vaccinated with past infection but no vaccine response or loss of vaccine-induced antibodies.
Positive	Positive	*More probably:* People vaccinated for COVID-19 with past SARS-CoV-2 infection. *Alternatively:* People not vaccinated with past SARS-CoV-2 infection.

COVID-19: coronavirus disease; N: nucleocapsid; S: spike; SARS-CoV-2: severe acute respiratory syndrome coronavirus 2.

## Statistical analysis

We estimated the anti-N and anti-S antibody seroprevalence in the whole study sample and by the characteristics of participants. The incidence of newly confirmed COVID-19 cases after the serological test was evaluated according to the antibody test result. Crude and adjusted odds ratios (aOR) with their 95% confidence intervals (CI) were calculated using logistic regression. Adjusted models included sex, age group (5–17, 18–29, 30–39, 40–49, 50–59, 60–69, 70–79 and ≥ 80 years), country of birth, major chronic conditions, previous confirmed COVID-19 and COVID-19 vaccination status. In the multivariate analysis of the risk of confirmed COVID-19, the history of confirmed COVID-19 and the vaccination status were not included because they can be expected to be closely associated with, respectively, anti-N and anti-S positive antibodies.

## Seroprevalence of antibodies against SARS-CoV-2

The study included a sample of 1,461 people from the population of Navarre aged 5 years and older. Anti-N antibodies were found in 860 individuals (58.9%). This seroprevalence decreased progressively with age from 85.1% in people aged 5–17 years to 26.0% in those aged 80 years and more, was higher in people born abroad (72.2%) and was lower in immunocompromised persons (29.3%). Among people with a previous diagnosis of confirmed COVID-19, the seroprevalence was 94.1%, and among those without such diagnosis, it was 38.0%. The multivariate analysis evidenced the strongest independent association with the previous diagnosis of COVID-19 (aOR = 22.10; 95% CI: 14.54–33.58). Older age, immunocompromised status, born in Spain and booster doses after completed primary vaccination were associated with lower seroprevalence of anti-N antibodies (Table 2).

**Table 2:** Factors associated with seroprevalence of anti-SARS-CoV-2 nucleocapsid protein antibodies, Navarre, Spain, 26 April–3 June 2022 (n = 1,461)

	Participants	Anti -N -positive		Crude analysis		Adjusted analysis	
	n	n	%	cOR	95% CI	aOR	95% CI
**Sex**							
Male	665	391	58.8	Reference		Reference	
Female	796	469	58.9	1.01	0.82–1.24	0.87	0.66–1.15
**Age (years)**							
5–17	181	154	85.1	Reference		Reference	
18–29	153	124	81.0	0.75	0.42–1.33	0.91	0.46–1.81
30–39	136	99	72.8	0.47	0.27–0.82	0.36	0.18–0.73
40–49	208	133	63.9	0.31	0.19–0.51	0.40	0.21–0.76
50–59	252	146	57.9	0.24	0.15–0.39	0.37	0.20–0.70
60–69	234	100	42.7	0.13	0.08–0.21	0.29	0.15–0.56
70–79	201	79	39.3	0.11	0.07–0.19	0.30	0.15–0.58
≥ 80	96	25	26.0	0.06	0.03–0.11	0.14	0.06–0.33
**Country of birth**							
Spain	1,274	725	56.9	Reference		Reference	
Other	187	135	72.2	1.97	1.40–2.76	1.73	1.11–2.69
**Major chronic conditions**							
No	839	536	63.9	Reference		Reference	
Immunocompromised	41	12	29.3	0.23	0.12–0.47	0.35	0.13–0.93
Other chronic condition	551	287	52.1	0.61	0.49–0.76	0.93	0.68–1.26
Unknown	30	25	83.3	NA		NA	
**Previous confirmed COVID-19**							
No	911	346	38.0	Reference		Reference	
Yes	525	494	94.1	26.02	17.68–38.31	22.10	14.54–33.58
Unknown	25	20	80.0	NA		NA	
**COVID-19 vaccination status**							
Unvaccinated	107	90	84.1	Reference		Reference	
Partial	64	59	92.2	2.23	0.78–6.37	0.50	0.15–1.70
Complete	404	338	83.7	0.97	0.54–1.73	0.70	0.36–1.35
Complete + one booster dose	778	339	43.6	0.15	0.09–0.25	0.33	0.17–0.62
Complete + two booster doses	89	18	20.2	0.05	0.02–0.10	0.18	0.07–0.41
Unknown	19	16	84.2	NA		NA	
**Total**	**1,461**	**860**	**58.9**				

aOR: adjusted odds ratio; CI: confidence interval; cOR: crude odds ratio; COVID-19: coronavirus disease; NA: not applicable (because categories with unknown values were not included in the models); SARS-CoV-2: severe acute respiratory syndrome coronavirus 2.

Anti-S antibodies were detected in 1,355 persons (92.7%). This seroprevalence was higher than 90% in almost all studied variables, but not in people aged 5–17 years (78.5%), with immunocompromised status (78.1%) and people not vaccinated against COVID-19 (39.3%). The COVID-19 vaccination was the unique variable independently associated with a higher seroprevalence of anti-S antibodies (Table 3).

**Table 3:** Factors associated to the seroprevalence of anti-SARS-CoV-2 spike protein antibodies, Navarre, Spain, 26 April–3 June 2022 (n = 1,461)

	Participants	Anti -S -positive		Crude analysis		Adjusted analysis	
	n	n	%	cOR	95% CI	aOR	95% CI
**Sex**							
Male	665	616	92.6	Reference		Reference	
Female	796	739	92.8	1.03	0.69–1.53	1.25	0.73–2.16
**Age (years)**							
5–17	181	142	78.5	Reference		Reference	
18–29	153	145	94.8	4.98	2.25–11.03	0.75	0.28–2.01
30–39	136	127	93.4	3.88	1.81–8.31	2.05	0.59–7.05
40–49	208	197	94.7	4.92	2.44–9.94	0.81	0.23–2.84
50–59	252	241	95.6	6.02	2.99–12.12	0.53	0.18–1.59
60–69	234	222	94.9	5.08	2.57–10.03	0.96	0.33–2.77
70–79	201	191	95.0	5.25	2.53–10.86	1.07	0.35–3.29
≥ 80	96	90	93.8	4.12	1.68–10.12	0.24	0.07–0.84
**Country of birth**							
Spain	1,274	1,186	93.1	Reference		Reference	
Other	187	169	90.4	0.70	0.41–1.19	2.09	0.88–4.95
**Major chronic conditions**							
No	839	780	93.0	Reference		Reference	
Immunocompromised	41	32	78.1	0.27	0.12–0.59	0.17	0.06–0.54
Other chronic condition	551	520	94.4	1.27	0.81–1.99	0.93	0.49–1.77
Unknown	30	23	76.7	NA		NA	
**Previous confirmed COVID-19**							
No	911	849	93.2	Reference		Reference	
Yes	525	487	92.8	0.94	0.62–1.42	1.74	0.94–3.23
Unknown	25	19	76.0	NA		NA	
**COVID-19 vaccination status**							
Unvaccinated	107	42	39.3	Reference		Reference	
Partial	64	61	95.3	31.47	9.27–106.85	31.80	8.89–113.70
Complete	404	395	97.8	67.92	31.57–146.15	77.09	34.22–173.67
Complete + one booster dose	778	767	98.6	107.91	53.03–219.60	208.28	82.09–528.47
Complete + two booster doses	89	78	87.6	10.97	5.23–23.02	32.31	10.87–95.98
Unknown	19	12	63.2	NA		NA	
**Total**	**1,461**	**1,355**	**92.7**				

aOR: adjusted odds ratio; CI: confidence interval; cOR: crude odds ratio; COVID-19: coronavirus disease; NA: not applicable (because categories with unknown values were not included in the models); SARS-CoV-2: severe acute respiratory syndrome coronavirus 2.

## Risk of confirmed COVID-19 by previous seroprevalence status

Among the 1,461 participants, 150 (10.3%) cases were confirmed for COVID-19 in the period from the date of the blood sample to 31 July 2022. In the bivariate analysis, the incidence of COVID-19 was higher among people negative for anti-N antibodies, those older than 18 years, born in Spain, without previous confirmed COVID-19, and completely vaccinated with booster doses.

In the multivariate analysis, anti-N antibody positivity were associated with a much lower risk of COVID-19 (aOR = 0.08; 95% CI: 0.05–0.13). Presence of anti-S antibodies (aOR = 1.06; 95% CI: 0.47–2.38) and all other variables were not statistically significantly associated with the risk of confirmed COVID-19. No interaction was found between anti-N and anti-S antibodies on risk of confirmed COVID-19 (p = 0.997) (Table 4).

**Table 4:** Analysis of the risk of COVID-19 since the sero-epidemiological survey, up to 31 July 2022, Navarre, Spain (n = 1,461)

	Participants	COVID-19 confirmed cases		Crude analysis		Adjusted analysis		
	n	n	%	cOR	95% CI	aOR		95% CI
**Anti-N antibodies**								
No	601	131	21.8	Reference		Reference		
Yes	860	19	2.2	0.08	0.05–0.13	0.08		0.05–0.13
**Anti-S antibodies**								
No	106	9	8.5	Reference		Reference		
Yes	1,355	141	10.4	1.13	0.62–2.53	1.06		0.47–2.38
**Sex**								
Male	665	61	9.2	Reference		Reference		
Female	796	89	11.2	1.25	0.88–1.76	1.24		0.86–1.80
**Age (years)**								
5–17	181	6	3.3	Reference		Reference		
18–29	153	14	9.2	2.94	1.10–7.84	2.87		1.00–8.24
30–39	136	12	8.8	2.82	1.03–7.72	2.15		0.73–6.34
40–49	208	19	9.1	2.93	1.14–7.51	1.52		0.55–4.16
50–59	252	31	12.3	4.09	1.67–10.03	1.90		0.72–4.98
60–69	234	27	11.5	3.80	1.54–9.43	1.28		0.48–3.41
70–79	201	27	13.4	4.53	1.82–11.23	1.39		0.51–3.74
≥ 80	96	14	14.6	4.98	1.85–13.42	1.19		0.40–3.50
**Country of birth**								
Spain	1,274	141	11.1	Reference		Reference		
Other	187	9	4.8	0.41	0.20–0.81	0.50		0.23–1.09
**Major chronic conditions**								
No	839	80	9.5	Reference		Reference		
Immunocompromised	41	4	9.8	1.03	0.36–2.95	0.61		0.20–1.84
Other chronic condition	551	65	11.8	1.27	0.90–1.79	1.08		0.73–1.62
Unknown	30	1	3.3	NA			NA	
**Previous confirmed COVID-19** ^a^								
No	911	130	14.3	Reference		Excluded		
Yes	525	19	3.6	0.23	0.14–0.37			
Unknown	25	1	4.0	NA				
**COVID-19 vaccination status** ^a^								
Unvaccinated	107	4	3.7	Reference		Excluded		
Partial	64	4	6.3	1.72	0.41–7.12			
Complete	404	24	5.9	1.63	0.55–4.79			
Complete + one booster dose	778	104	13.4	3.97	1.43–11.02			
Complete + two booster doses	89	13	14.6	4.40	1.38–14.04			
Unknown	19	1	5.3	NA				
**Total**	**1,461**	**150**	**10.3**					

aOR: adjusted odds ratio; CI: confidence interval; cOR: crude odds ratio; COVID-19: coronavirus disease; NA: not applicable (because categories with unknown values were not included in the models).

^a^ Previous confirmed COVID-19 and vaccination status were not included in the multivariate model because they were closely associated with, respectively, anti-N and anti-S antibody results and both lost statistical significance in the fully adjusted model.

## Discussion

By May 2022, 92% of the population of Navarre aged 5 years and older had anti-S antibodies, a percentage consistent with the 91% vaccination coverage in the region [4], and with the close association that we found between COVID-19 vaccination and the presence of these antibodies [10]. More than half of the population had anti-N antibodies, suggesting an immune response to SARS-CoV-2 infection. Among the participants in this survey, a history of confirmed COVID-19 was strongly associated with the presence of anti-N antibodies. In people with anti-N antibodies, the incidence of COVID-19 was greatly reduced, while anti-S antibodies did not modify this risk during the period of circulation of the Omicron BA.4 and BA.5 subvariants.

The seroprevalence of anti-N antibodies markedly decreased with age, as described in the United States [11]. Therefore, the older population remained, on average, more susceptible than the young people, and the relaxation of non-pharmacological preventive measures in the former group led to an increased risk of COVID-19, even though most of them were vaccinated. Immunocompromised people had a lower seroprevalence of anti-N and anti-S antibodies, probably due to poor immunological response and faster waning. Booster doses after complete primary COVID-19 vaccination were associated with a lower prevalence of anti-N antibodies i.e. natural infection, probably due to the effect of COVID-19 vaccines in preventing SARS-CoV-2 infection that was especially found against pre-Omicron variants in the first months after the last dose [12].

COVID-19 vaccination induces an immune response against one specific antigen (S-protein), while natural infection may induce a response against different antigens of the SARS-CoV-2, including the N-protein; therefore, a more powerful preventive response is expected [13,14]. Furthermore, while the antigen included in the vaccines considered the variants circulating in 2020, natural infection is an exposure to the antigens of the current circulating variants, which may be closer to future ones.

Vaccine-induced immunity has demonstrated high effectiveness in preventing severe COVID-19 outcomes regardless of the SARS-CoV-2 variant [15,16]. The present results show that vaccine-induced immunity has insufficient effect for preventing SARS-CoV-2 infections during the circulation of the Omicron BA.4 and BA.5 subvariants, while immunity induced by natural infection considerably reduces the risk of infection. Therefore, the combination of vaccine-induced and natural immunity provides more complete protection against SARS-CoV-2 infection and severe outcomes. Other authors have also reported that hybrid immunity from previous infection and recent booster vaccination conferred the strongest protection [1,2,13,14,17].

Since anti-N antibodies are induced by natural infection but not after COVID-19 vaccination, people who have not had COVID-19 remain highly susceptible to infection if exposed to the SARS-CoV-2 Omicron subvariants BA.4 and BA.5.

In the present study, the combined results of a seroprevalence survey and the enhanced surveillance of COVID-19 cases in the same region help to understand the new epidemic wave of the Omicron BA.4 and BA.5 subvariants in a region with high vaccination coverage. Although Navarre has an enhanced epidemiological surveillance of COVID-19, we found that 41.2% (346/840) of people with anti-N antibodies did not have previous confirmed COVID-19, suggesting that the healthcare system may have missed many SARS-CoV-2 infections.

This study has some limitations. Since it studied an opportunistic sample, the representativeness of the population of Navarre was not complete [9]. However, the results from our sample were consistent with those derived from epidemiological surveillance. Furthermore, the representativeness is not essential to establish the validity of the association between serological status and incidence of infection. The survey did not include nursing home residents, health professionals or children younger than 5 years, so the results would not apply to these groups. Initial antibody titres may have been lost over time in some participants. Analysis by vaccine brand was not considered due to the small sample size.

## Conclusion

The Omicron BA.4 and BA.5 subvariants of SARS-CoV-2 have circulated widely in a population with very high prevalence of anti-S antibodies after vaccination and where more than half of the people had anti-N antibodies. Hybrid immunity confers the strongest protection against COVID-19 because anti-N antibodies induced by natural exposure to SARS-CoV-2 seem essential to prevent SARS-CoV-2 infections, adding to the effect of vaccination to prevent severe COVID-19 outcomes. Since the prevalence of anti-N antibodies was lower in older people, they remained more susceptible to SARS-CoV-2 infection if exposed.
